# Financial capacity in patients with brain tumors: determinants and evolution after neurosurgery

**DOI:** 10.3389/fneur.2026.1774306

**Published:** 2026-04-15

**Authors:** Martina Andrea Sirtori, Francesca Colombo, Giorgia Repaci, Ilaria Mauri, Daniele Licciardo, Valeria Isella, Andrea Di Cristofori, Carlo Giorgio Giussani, Nadia Bolognini

**Affiliations:** 1 Neurosurgery Department, IRCCS Humanitas Research Hospital, Rozzano, Milan, Italy; 2 Department of Psychology, University of Milano-Bicocca, Milan, Italy; 3 Postgraduate School of Psychology Agostino Gemelli (ASAG), Università Cattolica del Sacro Cuore, Milan, Italy; 4 Neurology Unit, Neuropsychology Center, Fondazione IRCCS San Gerardo dei Tintori, Monza, Italy; 5 Division of Neurosurgery, Fondazione IRCCS San Gerardo dei Tintori, Monza, Italy; 6 School of Medicine and Surgery, University of Milano-Bicocca, Milan, Italy; 7 Laboratory of Neuropsychology, Department of Neurorehabilitation Sciences, IRCCS Istituto Auxologico Italiano, Milan, Italy

**Keywords:** brain tumor, cognitive deficits, financial capacity, neuropsychological assessment, neurosurgery

## Abstract

**Introduction:**

Financial capacity (FC) is a functional ability, mediated by cognitive functions, that is vulnerable to neurological conditions; its decline negatively affects independence in daily life. This study aimed to explore FC impairments and their evolution in patients with neoplastic brain lesions, taking into account: the influence of lesion and socio-demographic characteristics and cognitive functioning on FC; the relationship between impaired FC and functional independence, also considering patients’ awareness of their own financial skills and that of their caregivers.

**Methods:**

A prospective observational design was adopted; 21 patients with brain tumors and 21 age- and education-matched healthy controls were enrolled at a neuro-oncology center. FC was evaluated using the standardized Numerical Activities of Daily Living—Financial (NADL-F) test. Cognitive functioning was screened with the Montreal Cognitive Assessment (MoCA) and the Frontal Assessment Battery (FAB); functional independence was assessed with the Instrumental Activities of Daily Living (IADL) scale.

**Results:**

Before neurosurgery, patients exhibited significantly lower total NADL-F scores compared to controls. The most pronounced FC-related deficits were observed in reading abilities and item purchase tasks. After tumor resection, 33% of patients showed improvement in FC, whereas 24% patients showed a further decline of their performance in financial tasks. FC was associated with lesion size and the presence of cognitive deficits (MoCA and FAB scores). Patients consistently overestimated their FC, as did their caregivers.

**Conclusion:**

Financial capacity appears to be substantially compromised in patients with brain tumors, with the degree of impairment correlating with tumor volume and cognitive deficits and worsened by a lack of awareness of the difficulties in the financial domains. The post-surgical outcomes are variable, with complete and partial recovery or further deterioration of FC. These results underscore the importance of including a systematic assessment of FC in the neuro-oncological evaluation process.

## Introduction

Financial capacity (FC) is conceptualized as a medico-legal construct that represents the ability to independently manage one’s financial affairs in a manner consistent with personal interests and values; it is essential to function independently in everyday life (1). This functional capacity encompasses a wide range of skills, from more basic and elementary abilities, such as handling cash and performing simple calculations during everyday shopping, to more complex competencies and actions, such as making informed decisions about financial investments and assets. It also pertains the ability to evaluate and then make financial decisions in line with one’s life goals, beliefs, and personal values (1, 2).

Over time, various conceptual models have been proposed to outline this construct (3–7), currently recognized as multidimensional (1). The modern conceptualization of FC (6) proposes a hierarchical division across three levels of increasing complexity, from the most basic and pragmatic skills (e.g., counting money, withdraw cash from an ATM) to knowledge and mastery of financial concepts (e.g., know what a bank transfer or a loan is), up to financial decision-making and judgment. This hierarchical structure has prompted the development of ad-hoc tests and questionnaires for a comprehensive assessment of FC, enabling clinicians to systematically assess specific financial skills, in turn identifying FC-related deficits for rehabilitation purposes in clinical settings, or for legal reasons in forensic contexts.

From a cognitive perspective, three fundamental FC components have been recognized: declarative knowledge, procedural knowledge, and judgment (3, 8–10). Declarative knowledge encompasses both episodic and semantic information related to financial concepts (for example, the ability to name different coins or to comprehend a bank statement). Procedural knowledge concerns knowledge of how to carry out financial activities, in terms of single actions and routines that, depending on daily use, are largely automated. Finally, judgment concerns the capacity to make informed and conscious choices, being able to evaluate and predict the consequences of one’s financial decisions (3). All these different components rely on distinct cognitive processes. For example, the ability to calculate the appropriate amount of money—such as when giving change or purchasing a product or to arrange coins by value—depends on arithmetic calculation abilities and knowledge of currency values (11–13). Visual-motor speed, instead, is implicated in tasks that involve rapid recognition and identification of currency, or the efficient inspection of banking information (13, 14). In addition, appropriate declarative or conceptual knowledge about financial concepts requires adequate memory capabilities (12, 13, 15), while efficient executive and attentive functioning is essential for the correct execution and monitoring of procedural skills (3, 16–18). Finally, executive functions and social cognition are essential for efficient financial decision-making, including fraud detection (16).

Beyond the cognitive underpinnings, psycho-social factors are also relevant in shaping individual FC and their role should not be underestimated. For instance: apathy and negative affect have been associated with impaired FC (19); depressive symptoms may increase the risk of financial exploitation (20), just as negative perceptions of one’s own psychological well-being (21), loneliness and poor social and familiar support increase the risk of being scammed (22).

Since FC depends largely on cognition, it is not surprising its vulnerability to aging and neurological or psychiatric diseases, and more generally to all medical conditions that affect mental functions (23). In particular, with respect to neurological patients, it has been well documented the decline of FC in neurodegenerative diseases such as Alzheimer’s Disease (24), Parkinson’s Disease (25, 26) and behavioral variant of Fronto-Temporal Dementia (27). The deterioration of FC emerges from the onset of dementia, even in its pre-clinical phase, as a marker of Mild Cognitive Impairment (13, 14, 28). Evidence of impaired FC has also been found in multiple sclerosis (29, 30), traumatic brain injury (18) and stroke (31).

However, to date, research on FC has focused mainly on healthy and pathological aging, while the study of FC and its evolution in patients with brain tumors has been largely neglected. To address this gap, the present study aimed at exploring: (1) FC in patients with neoplastic brain lesions and its evolution over time, particularly from the pre-surgical to the post-surgical phase; (2) the relationship between FC and tumor-related features, especially the lesion location and volume, the presence of edema and its histological characteristics; (3) the associations between FC and cognitive functioning, and FC awareness as reported by patients and their caregivers.

## Materials and methods

### Participants

Twenty-one patients with intra- or extra-axial brain neoplasms were enrolled at the Neurosurgery Unit of San Gerardo Hospital in Monza, along with 21 healthy control subjects (see Table 1 for socio-demographic and clinical data). Inclusion criteria for patients were as follows: (1) age over 20 years, (2) a clinical diagnosis of glioma or meningioma, subsequently confirmed through histological examination, and (3) undergoing neurosurgical resection of the lesion.

**Table 1:** Socio-demographic characteristics of clinical and control groups; lesion-related variables of the patient group.

Features	Patients (*N* = 21)	Controls (*N* = 21)
Age, mean (Standard Deviation, SD)	58.3 (10.2)	58.3 (12.1)
Gender, female/male	9 (43%)/12 (57%)	10 (48%)/11 (52%)
Education, mean (SD)	11.9 (3.3)	13.3 (3.55)
Handedness, right (%), ambidextrous (%), left (%)	20 (95%), 1 (5%), 0	17 (81%), 3 (14%), 1 (5%)
Brain tumor, meningioma (%), glioma (%)	7 (33%), 14 (67%)	/
Grade, I (%), II (%), IV (%)	7 (33.3%), 3 (14.3%), 11 (52.4%)	/
Side, left (%), right (%), bilateral (%)	6 (28.6%), 12 (57.1%), 3 (14.3%)	/
Location, anterior (%), posterior (%)	11 (52%), 10 (48%)	/
Oedema, present (%), absent (%)	11 (52%), 10 (48%)	/

All patients underwent neurosurgery to remove the tumor mass, with pre- and post-operative brain Magnetic Resonance Imaging (MRI), as a validated standard neurosurgical procedure. Neurosurgical complication, consisting of a mild hemorrhagic lining of the surgical cavity, occurred in only one case. None of the patients with meningioma underwent adjuvant postoperative treatment. Among the patients with glioma who completed the follow-up assessment (see below), one patient with a grade I ganglioglioma did not receive any adjuvant therapy, one patient with a grade II oligodendroglioma received chemotherapy alone, and 8 patients with grade IV gliomas received combined radiotherapy and chemotherapy.

Patients and control participants with a history of psychiatric disorders, neurological diseases (in addition to the current tumor in the case of patients), or significant sensory-motor or language impairments that could compromise the reliability of the assessment using the tests covered by this study (see below) were excluded.

All participants received detailed information regarding the study procedures and voluntarily agreed to participate by signing a written informed consent form. Data were handled in accordance with the guidelines of the Declaration of Helsinki and the Oviedo Convention (latest revision, 2014). The study was approved by the Ethics Committee of the Istituto Auxologico Italiano (protocol number: 25C121-NEUROFIN).

### Study design and neuropsychological assessment

Patients underwent an assessment of FC and cognitive functioning in two sessions: before surgical resection (T0) and 3 months after surgery (T1). Healthy controls were subjected to a single session of assessment. Each session lasted approximately 1 h and was conducted in a quiet, well-lit room to minimize environmental distractions.

The neuropsychological assessment consisted of screening for global cognitive and executive functioning using the Montreal Cognitive Assessment (MoCA) (32), the Frontal Assessment Battery (FAB) (33), respectively, and the evaluation of functional independence in daily life using the IADL scale (6).

FC was assessed by administering the Numerical Activities of Daily Living-Financial [NADL-F; (34)], a validated and standardized battery of 7 tasks that simulate real-life financial activities, covering several domains in accordance with current theoretical models of FC (8). In particular, the NADL-F comprises the following subtests [for a detailed description of the task, see (34)]:

1. Counting currencies, which assesses familiarity with currency and the ability to perform mental calculations using money, similar to those involved in simple cash transactions;
2. Reading abilities to evaluate the capacity to deal with written information about money involved in everyday life situations;
3. Item purchase, which evaluates the ability to manage transactions required for simulated monetary exchanges, involving product purchases and change verification;
4. Percentages, a task that assesses the ability to perform mental calculations in percentages required in real-life situations;
5. Financial concepts to assess knowledge of relevant financial concepts;
6. Bill payments, which assesses skills in bill management;
7. Financial judgments, which explores financial decision-making and recognition of fraudulent behaviors.

Scores at every subtest were adjusted for age, education, and, if applicable, gender, and then classified as either “within normal limits” or “impaired,” according to the respective cut-off scores. The total NADL-F score is then computed as an unweighted sum of the individual subtest scores and serves as a proxy for overall FC severity. There is no formal normative threshold (cut-off value) for the total score.

The NADL-F also includes a semi-structured interview to assess the awareness of the individual’s financial capacity, with different questions reflecting the same skills evaluated in the NADL-F tasks. The interview is administered to both the patient and his/her caregiver. Its score ranges from 0 to 22, with a score of 22 indicating the perception of full preservation of FC. Differences between the two evaluations made by the patient and the caregiver is used to derive a FC awareness index: the awareness index score range from 0 to 22, 0 reflecting agreement in reported FC between raters, with positive and negative values reflecting, respectively, overestimation and underestimation by the patient of the own FC (34).

### Statistical analysis

Statistical analyses were performed using Jamovi (version 2.4; The jamovi project, 2023). Due to violations of normality assumptions, even after data transformation, non-parametric statistics were employed.

Our primary outcome was the total NADL-F score, whereas subtests scores were secondary endpoint, to be considered in exploratory terms.

Group differences between patients and healthy controls regarding age and education were assessed using Mann–Whitney U tests, while the gender distribution was compared using the Chi-square test. Between-group (patients vs. controls) differences in NADL-F performance were analyzed using the Mann–Whitney *U* test, separately for T0 and T1. Since healthy controls were tested once, and their scores were compared to those of patients at both time-points (T0 and T1). Differences in subtest outcomes (normal vs. impaired) across groups were assessed using Chi-square tests.

Within the clinical group, differences over time (T0 vs. T1) in every NADL-F subtest and in the total NADL-F score were tested using Friedman’s ANOVA. Spearman’s rank correlation analyses were then conducted to examine the relationship between global NADL-F score and tumor characteristics and patient’s educational level. To explore associations between FC and cognitive functioning, Spearman’s rank correlations were performed between NADL-F total score and the MoCA, FAB and IADL scores. The Mann–Whitney *U* test or the Kruskal-Wallis test, as appropriate, were used to evaluate differences in total NADL-F scores according to lesion profile (e.g., laterality, histology, location, edema) at both timepoints (T0 and T1).

Finally, the association between FC (total NADL-F score) and its awareness (FC awareness index) was explored by Spearman’s rho correlation, and discrepancies were analyzed qualitatively.

## Results

Five out of 21 recruited patients dropped out at the T1 assessment: 2 of them because they started palliative care, while the other 3 patients continued their treatment at another hospital, discontinuing follow-up evaluations. Hence, statistical analyses considering performance at T1 were conducted on 16 out of 21 patients with brain tumors.

### The FC of patients with brain tumors compared to healthy controls

No significant differences were observed between the clinical and control groups in terms of age (U = 214, *p* = 0.88), education (U = 167, *p* = 0.17), or gender distribution (χ^2^ = 0.10, *p* = 0.76).

**Figure 1:**
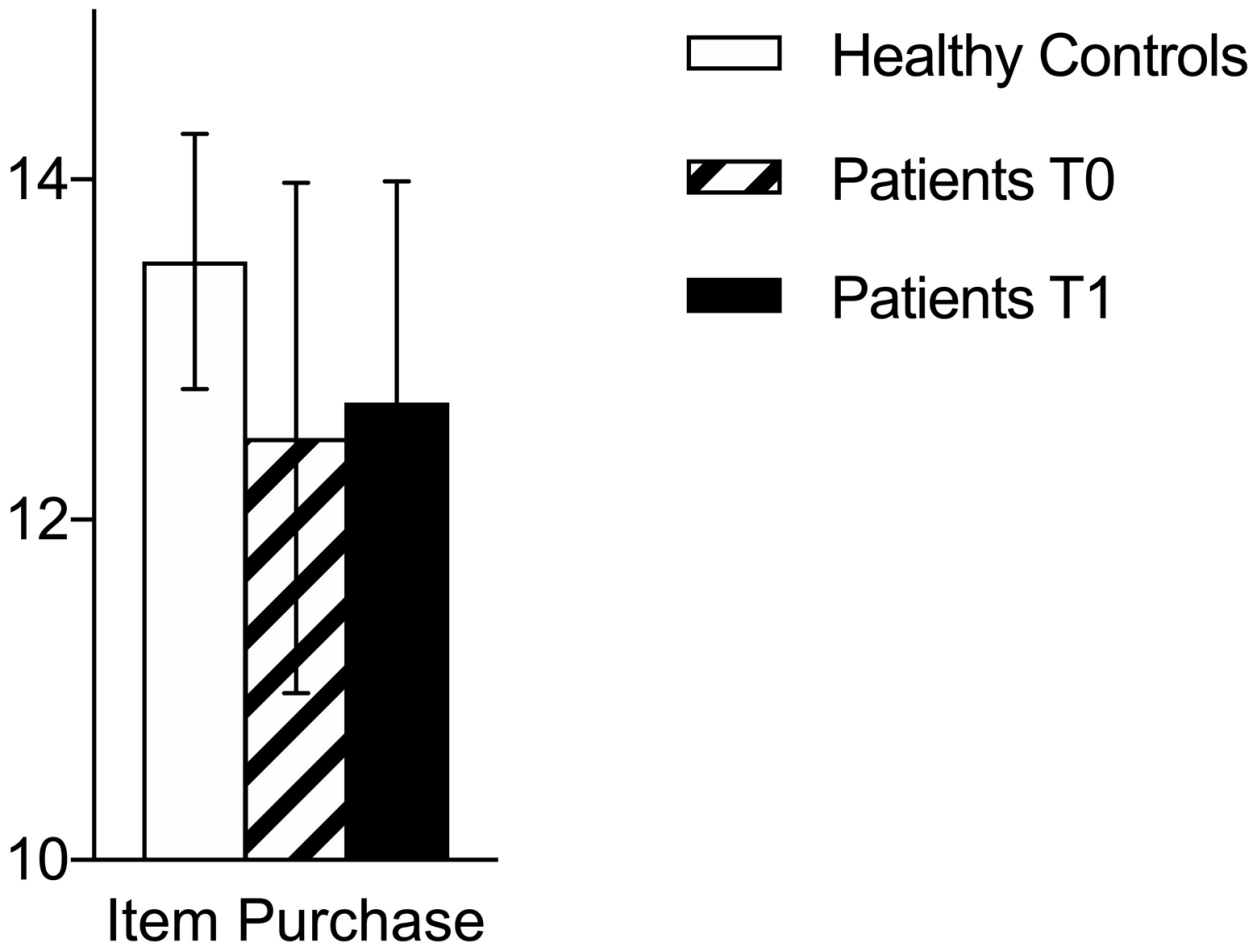
Comparison between clinical and control group on NADL-F Item Purchase subtest at T0 and T1.

**Figure 2:**
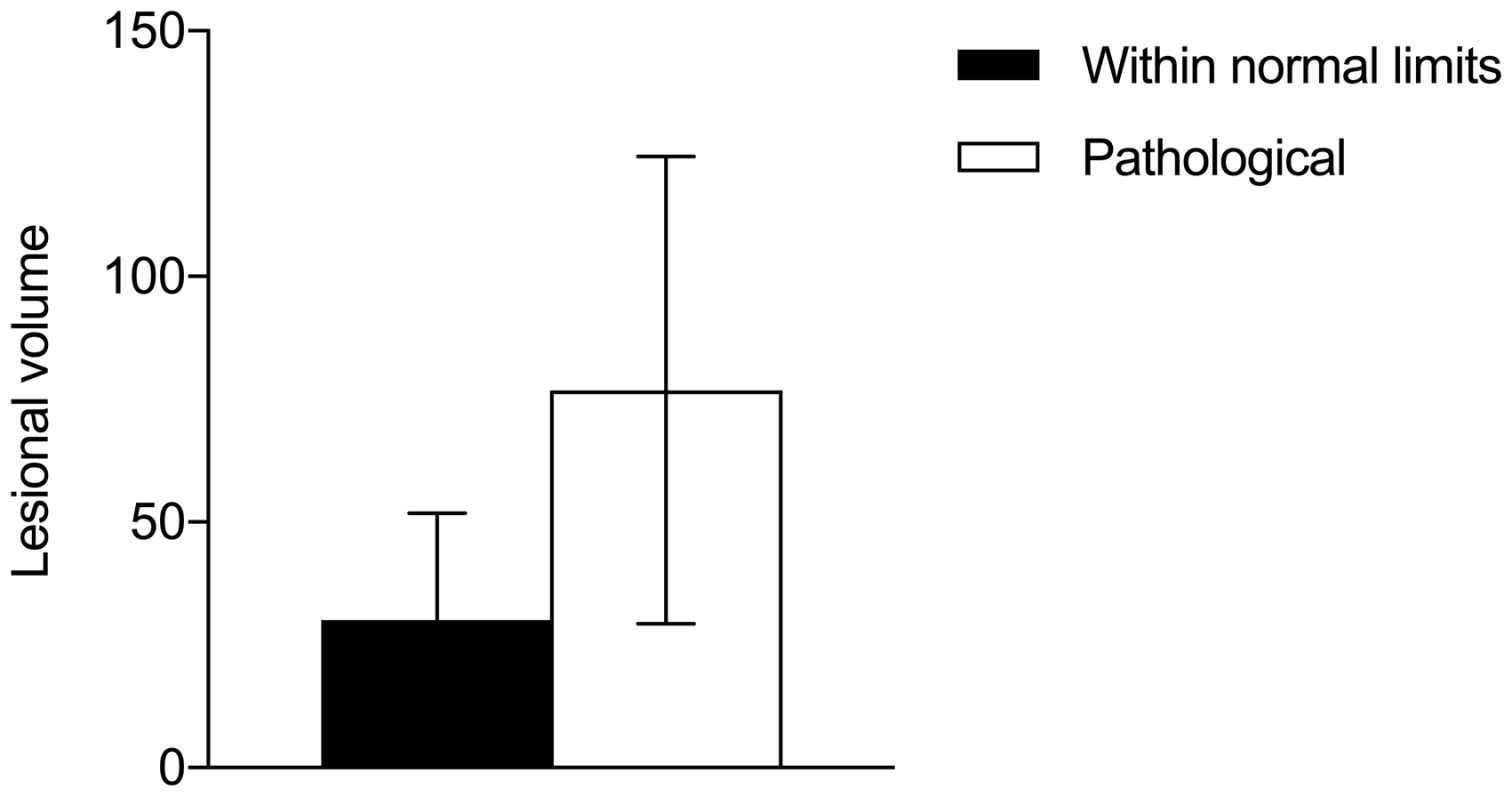
Comparison of lesion volume between patients with normal or impaired FC.

**Figure 3:**
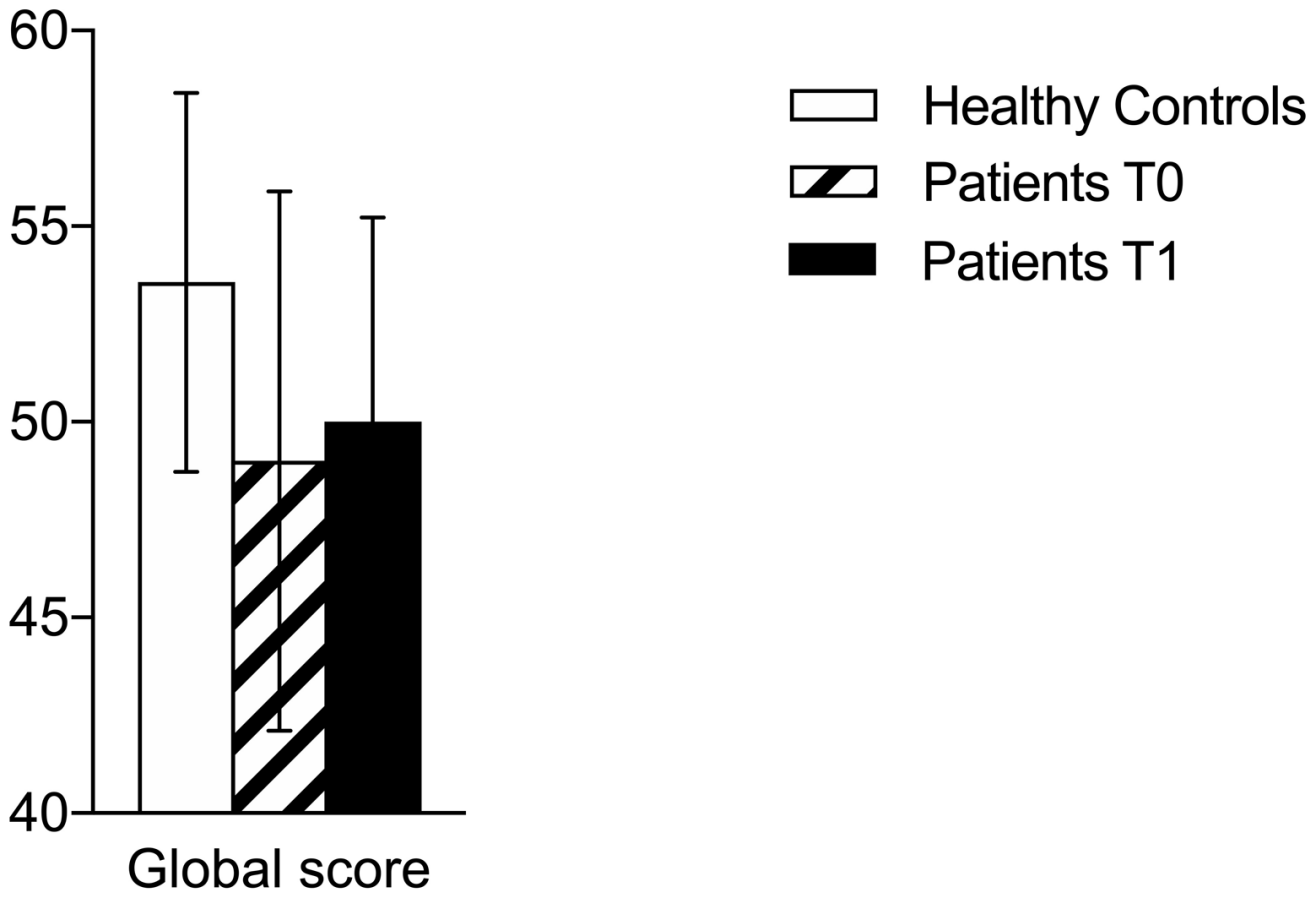
Comparison between clinical and control group on NADL-F global score at T0 and T1.

At T0, patients with brain tumors showed reduced FC (total NADL-F score) than healthy controls (patients = 49.0 vs. healthy controls = 53.57, U = 129, *p* = 0.021). Patients scored lower in the following NADL-F subtests (see Table 2): Counting currencies (U = 147, *p* < 0.01), Reading abilities (U = 157, *p* = 0.019), Item purchase (U = 128, *p* = 0.012), and global score (U = 129, *p* = 0.021; See Figures 1–3). A similar trend emerged at the follow-up (T1; see Table 2): total NADL-F score (patients = 50.0 vs. healthy controls = 53.57; U = 97.5, *p* = 0.03), Reading abilities (U = 123, *p* = 0.033), Item purchase (U = 99, *p* = 0.022).

**Table 2:** Means (M) and standard deviations (SD) for NADL-F domains and global score.

NADL-F	Controls, M (SD)	Lower/upper CIs	Patients T0, M (SD)	Lower/upper CIs	*p*-value	Patients T1, M (SD)	*p*-value	Lower/upper CIs
Total NADL-F score	53.57 (4.84)	51.4–55.8	49.0 (6.89)	45.9–52.1	0.021*	50.0 (5.23)	0.031*	47.2–52.8
Counting currencies	5.00 (0)	5.00–5.00	4.43 (1.08)	3.94–4.92	0.005*	4.81 (0.54)	0.109	4.52–5.10
Reading abilities	7.95 (0.22)	7.85–8.05	7.52 (0.81)	7.15–7.89	0.019*	7.63 (0.62)	0.033*	7.30–7.95
Item purchase	13.52 (0.75)	13.2–13.9	12.48 (1.50)	11.8–13.2	0.012*	12.69 (1.30)	0.022*	12.0–13.4
Percentages	7.33 (1.96)	6.44–8.22	6.19 (2.34)	5.13–7.25	0.126	6.44 (2.31)	0.209	5.21–7.67
Financial concepts	8.57 (2.36)	7.50–9.64	8.19 (2.50)	7.05–9.33	0.759	7.69 (2.27)	0.187	6.48–8.90
Bill payments	5.43 (0.81)	5.06–5.80	4.86 (1.15)	4.33–5.38	0.081	5.13 (0.62)	0.129	4.80–5.45
Financial judgments	5.76 (0.44)	5.56–5.96	5.33 (1.06)	4.85–5.82	0.205	5.63 (0.62)	0.565	5.30–5.95

Significance of comparisons between control and patient’s groups are presented at. T0 (i.e., pre-operative) and T1 (i.e., follow-up). *statistically significant values, with *p* value set at < 0.05, comparing clinical and control groups.

Considering the number of participants scoring who scored below the cut-off values on the NADL-F subtests (34), at T0 patients showed a significantly higher frequency of impaired performance only in Financial Judgments compared to controls (χ^2^ = 4.42, *p* = 0.035). No other subtests showed differences in scores at either T0 or T1.

However, when patients were considered separately according to the lesioned hemisphere, patients with right-sided hemispheric lesions performed, at both assessments, significantly worse than controls in the following subtests: Counting currencies (T0: U = 94.5, *p* = 0.02, *r* = 0.25; T1: U = 84, *p* = 0.042, *r* = 0.20), Reading abilities (T0: U = 89.5, *p* = 0.03, *r* = 0.29; T1: U = 67.5, *p* = 0.014, *r* = 0.36), and Item purchase (T0: U = 74, *p* = 0.034, *r* = 0.41; T1: U = 54.5, *p* = 0.019, *r* = 0.48). At T1, right-sided brain-damaged patients differed from controls also with respect to the total NADL-F score (U = 53, *p* = 0.029, *r* = 0.49).

Instead, patients with left-sided hemispheric lesions differed from controls only in Financial judgments and only at T0 (U = 31, *p* = 0.027, *r* = 0.51).

### Evolution of FC in patients with brain tumors

Considering only the group of patients who underwent the longitudinal assessment of FC, the Friedman’s ANOVA revealed no significant effect of Time (T0 vs. T1) both in terms of the total NADL-F score (χ^2^ = 0.07, *p* = 0.796) and the scores for its subtests: Counting currencies: χ^2^ = 1.00, *p* = 0.317; Reading abilities: χ^2^ = 0.14, *p* = 0.705; Item purchase: χ^2^ = 0.00, *p* = 1.00; Percentages: χ^2^ = 0.08, *p* = 0.782; Financial concepts: χ^2^ = 1.67, *p* = 0.197; Bill payments: χ^2^ = 0.50, *p* = 0.480; Financial judgments: χ^2^ = 0.67, *p* = 0.414.

Patients’ total NADL-F score was not influenced by presence of edema (T0: U = 30.5, *p* = 0.088), lesion location (T0: U = 49.5, *p* = 0.723; T1: U = 24, *p* = 0.426), histology (T0: U = 30.5, *p* = 0.176; T1: U = 27, *p* = 0.784), or lesion laterality (T0: H = 2.37, *p* = 0.306; T1: H = 1.40, *p* = 0.497). A significant correlation was found between patients’ level of education and the pre-operative total NADL-F score (*ρ* = 0.60, *p* = 0.004), whereas no significant correlation was observed with the post-operative score (*ρ* = 0.18, *p* = 0.51).

Considering the pre-surgical assessment (T0), the comparison between patients with impaired performance in one or more financial subtests and those with normal scores in all the NADL-F subtests, showed no differences in relation to the edema (χ^2^ = 0.687, *p* = 0.407), lesion location (χ^2^ = 0.69, *p* = 0.407), histology (χ^2^ = 1.05, *p* = 0.306), or lesion laterality (χ^2^ = 2.63, *p* = 0.269).

**Figure 4:**
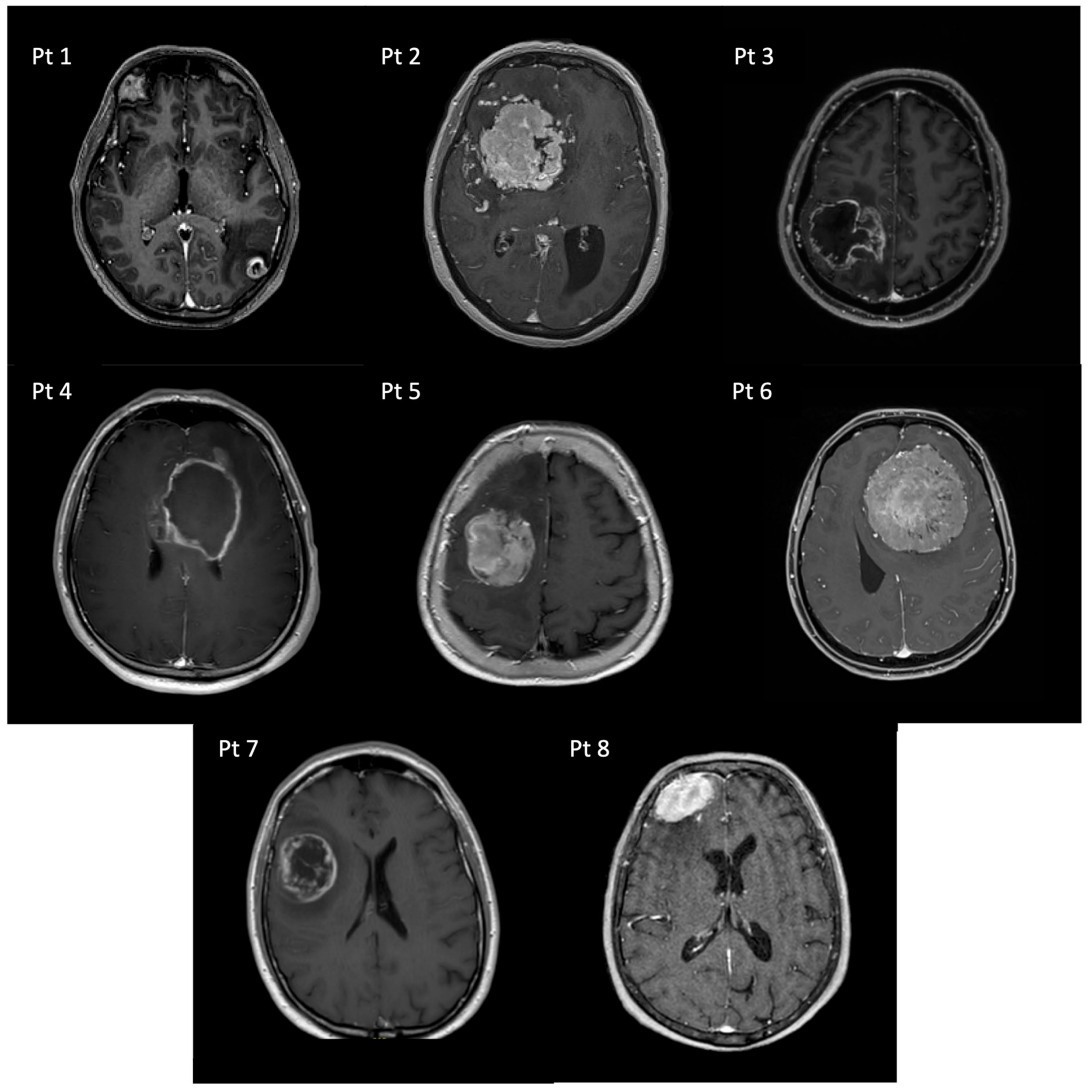
Pre-operative axial T1-weighted MRI scans of patients with impaired FC.

Instead, the lesion volume had an effect on FC (see Figure 4): at T0 but not at T1, impaired patients presented with larger tumors (U = 12, 95% CI: 6.10–85.3, *p* = 0.011) compared to unimpaired patients, even though no significant correlation was found overall between lesion volume and the total NADL-F score (T0: *ρ* = −0.19, *p* = 0.401; T1: ρ = 0.07, *p* = 0.797). Considering patients with temporal lesions, tumor volume did not correlate with total NADL-F scores at neither T0 (ρ = 0.29, *p* = 0.577) nor T1 (ρ = 0.03, *p* = 0.949). The same results were found for patients with frontal lesions (T0: ρ = −0.38, *p* = 0.252; T1: ρ = 0.04, *p* = 0.932).

Pre-operative total NADL-F score correlated significantly with FAB (ρ = 0.61, *p* = 0.003) and MoCA (ρ = 0.64, *p* = 0.002) scores, but no significant correlation emerged with IADL scores (ρ = 0.07, *p* = 0.761). At follow-up, only the correlation between MoCA and total NADL-F scores remained significant (ρ = 0.59, *p* = 0.016; FAB: ρ = 0.42, *p* = 0.104; IADL: ρ = 0.26, *p* = 0.331). No correlations were observed between Financial judgments and neither FAB (T0: ρ = 0.27, *p* = 0.231; T1: ρ = −0.21, *p* = 0.425) nor MoCA (T0: ρ = 0.21, *p* = 0.357; T1: ρ = 0.32, *p* = 0.907).

### FC awareness

At T0, no significant correlation was found between the total NADL-F score and the self-evaluation of FC (ρ = 0.23, *p* = 0.322) or the caregiver evaluation of the patient’s FC (ρ = 0.40, *p* = 0.074); the same occurred at T1 (self-evaluation: ρ = 0.36, *p* = 0.171; caregiver: ρ = 0.24, *p* = 0.376). Only at T0 the FC evaluations of FC of patients and caregivers significantly correlated (ρ = 0.55, *p* = 0.009; T1: ρ = 0.48, *p* = 0.062).

### Qualitative observations

Prior to surgery (T0), the 29% (6 out of 21) patients with brain tumors showed impaired performance in at least one NADL-F subtest. This percentage dropped to 12% (2 out of 16) at post-surgical follow-up. Fifty percent of patients who were impaired (3 out of 6) before tumor removal showed full recovery of all the financial skills measured by the NADL-F subtests. One patient showed partial recovery, showing at T1 an unimpaired performance in 3 subtests, with persistent impaired performance in financial concepts. Unfortunately, two patients presenting with a pathological performance at T0 did not undergo post-surgical evaluation. Finally, there were 2 patients impaired at T1, but not at T0.

**Figure 5:**
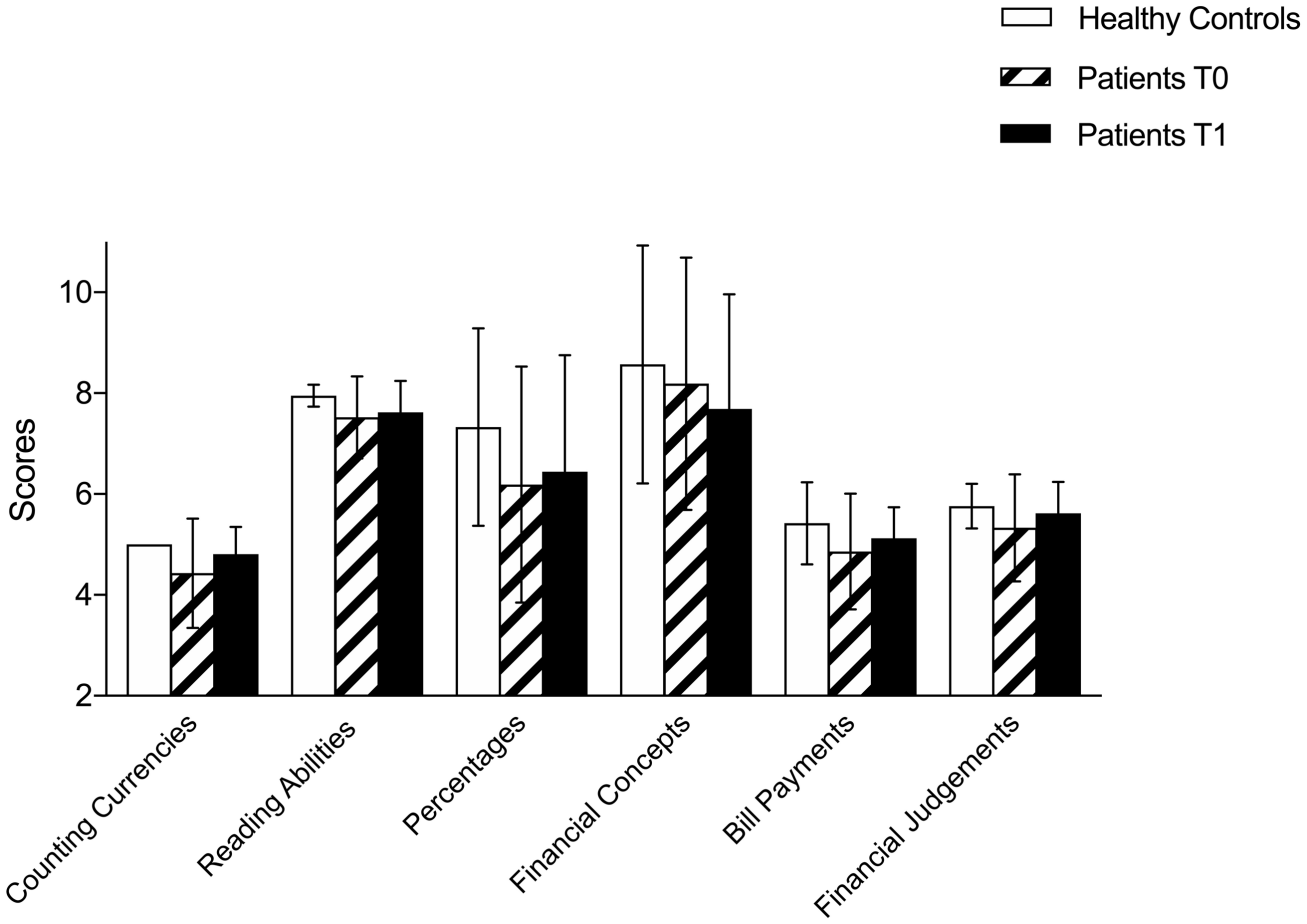
Comparison between clinical and control group on NADL-F domains’ scores at T0 and T1.

Noteworthy, patients presenting with impaired FC had heterogeneous lesion characteristics (see Table 3; Figure 5 for details): they had both intra- or extra-axial tumors, they differ with respect to their lesion volume, they had or not edema. Four out of six patients with impaired FC at T0 had anterior lesions, but with no apparent hemispheric dominance. Both patients impaired at T1 had right frontal lesions. Only one patient scored pathologically on the FAB before surgery, while none showed deficits on the MoCA. With respect to the two cases that showed post-operative deterioration in FC, one of them also had a concomitant decline in executive and global cognitive functioning, while the other patient showed only a slight decline on the FAB.

**Table 3:** Lesional and cognitive variables of patients with at least one score below the cut-off on the NADL-F at T0 (i.e., pre-operative) and T1 (i.e., follow-up).

Patient	Histology	Volume	Edema	Location	Side	Total NADL-F score		Impaired NADL-F subtest		MoCA		FAB		ADL		IADL	
						T0	T1	T0	T1	T0	T1	T0	T1	T0	T1	T0	T1
Pt 1	Glioma	22.6	Yes	Posterior	Left	37	45	Bill Payments, Financial Judgments, Reading ability, Percentages	-	23.11	21.11	15.3	17.3	6	6	6	6
Pt 2	Meningioma	103	No	Anterior	Right	42	55	Counting currencies, Item purchase, Bill Payments	-	18.52	23.52	15.8	15.8	2	6	7	8
Pt 3	Glioma	79	Yes	Posterior	Right	42	48	Financial concepts, Bill Payments, Financial Judgments	Financial concepts	20.52	24.52	15.9	15.9	6	6	8	8
Pt 4	Glioma	66.4	Yes	Anterior	Bilateral	36	N/A	Counting currencies, Financial concepts, Percentages, Bill Payments	N/A	19.11	N/A	**9.4**	N/A	6	N/A	7	N/A
Pt 5	Meningioma	36.4	Yes	Anterior	Right	41	43	Financial concepts	-	21.72	22.72	*13.9*	14.9	6	6	8	6
Pt 6	Meningioma	153.9	No	Anterior	Bilateral	44	N/A	Item purchase, Financial Judgments	N/A	21.15	N/A	15.9	N/A	5	N/A	3	N/A
Pt 7	Glioma	30.6	Yes	Anterior	Right	57	42	-	Counting currencies, Percentages, Item purchase, Financial concepts	23.98	*17.98*	18	**12**	6	6	8	6
Pt 8	Meningioma	13.2	Yes	Anterior	Right	46	43	-	Percentages	22.58	20.58	14.7	*13.7*	6	6	8	8

ADL, activities of daily living; FAB, frontal assessment battery; IADL, instrumental activities of daily living; MoCA, Montreal Cognitive Assessment; N/A not applicable; Pt, patient. Bold type, pathological score; Italic, scores at lower normative limits.

With respect to FC awareness, among the six patients with at least one impaired FC domain at T0, four of them overestimated their capacity, scoring the maximum of 22/22 at the FC interview. Caregivers overestimated the FC of four of these six impaired patients. At T1, patients and the caregivers of the two impaired individuals overestimated the performances.

## Discussion

The current study explored the integrity of FC in a cohort of patients with neoplastic brain lesions (glioma and meningioma), as compared to healthy controls, also tracking changes in FC from pre- to post-surgery (i.e., 3 months after the neurosurgical resection). Our findings, although preliminary given the small sample size, suggest that patients with brain tumors exhibit significant, although selective, deficits in FC, which may recover after the tumor removal, although a vulnerability may also emerge after the surgical intervention. FC impairments are compounded by compromised awareness of these functional impairments.

In particular, patients with brain tumors obtained significantly lower NADL-F total scores compared to age-matched healthy controls, both pre-operatively and 3 months after surgery, hence documenting a diminished FC in this clinical group. Particularly, patients with brain tumors exhibited reduced capacities in financial abilities encompassing those required by some NADL-F tasks such as Counting Currencies, Reading Abilities and Item Purchase, compared to controls. These tasks reflect everyday financial skills and require the integration of both conceptual financial knowledge (e.g., understanding monetary values and written financial information) and procedural abilities (e.g., carrying out simple transactions).

These selective decline in some financial skills, however, did not reach a pathological level: patients’ scores at each NADL-F subtest were indeed within a normal range according to the Italian norms and related cut-off values (34), although being lower compared to the scores obtained by healthy individuals. This finding is partly consistent with the results reported by a study in stroke patients (31), who observed a selective reduction in basic components of FC, whereas more complex financials skills appeared relatively preserved. Along with the present work, the pattern of results suggests that neurological conditions characterized by focal (vascular or neoplastic) lesions may initially affect the more elementary components of financial competence, while leaving higher-level financial abilities relatively intact. However, an important methodological difference should be highlighted. Danesin and colleagues did not consider the clinical relevance of the scores with respect to normative cut-off values. By contrast, in the present study patients’ performances were interpreted in relation to established normative thresholds, allowing a clearer distinction between a mere reduction in performance and a clinically significant deficit. Consistently with this normative approach, a pathological impairment was detected only in a single NADL-F subtest, the Financial Judgments in the pre-operative phase, with scores returning to normal levels at follow-up. These results suggest that while patients exhibit a reduction in FC compared to healthy controls, it does not always manifest as a clinically relevant impairment, except for a specific vulnerability in financial judgment that may recover after brain surgery.

It is also important to note that, although education is known to influence financial capacity (29), and was correlated with patients’ global NADL-F score at baseline, the clinical and control groups did not differ in educational level; therefore, the FC decline observed in patients with brain tumors cannot be attributed to differences in years of schooling.

Of interest is also the qualitative analysis of individual patient performance, from which it emerges the complete or partial recovery of FC impairments between the pre-operative assessment and the 3-months, post-surgical, follow-up. Specifically, while 29% of patients exhibited impairments in at least one domain of financial capacity prior to surgery, only 12% showed significant difficulties at the follow-up. Notably, there is several cases, albeit small, showing a decline of FC after tumor removal surgery, as observed at the 3-months follow-up, even in the absence of any deficit prior to tumor resection. These findings are of considerable interest, as they highlight, on the one hand, the potential for functional recovery of FC following the resection of a brain lesion, but also the need of longitudinal assessment also to detect the emergence of post-surgery impairments. These results are consistent with existing literature on cognitive outcomes following brain tumor resection, which generally reports a substantial recovery of function within 3 months post-surgery, alongside a smaller proportion of patients who experience cognitive deterioration following surgery (35, 36).

The patient who showed the most pronounced impairment at the three-month follow-up also exhibited a broader clinical deterioration. In addition to the worsening in FC, this patient showed a decline in global cognitive (MoCA) and executive (FAB) functioning, and functional independence in instrumental activities of daily living (IADL). This pattern suggests that a marked reduction in FC may occur in the context of a more generalized cognitive and functional decline, as previous evidence shows (3).

With respect to the lesion profile of our patients, we found no association between their FC and their lesion-characteristics. In particular, the lesion volume did not correlate with the total NADL-F score. However, a more subtle relationship between the lesion volume and financial skills emerged: patients with at least one pathological score at the NADL-F subtests had significantly larger lesions compared to those with more localized lesions. Nonetheless, pathological scores at the NADL-F subtests were also observed in patients with relatively confined lesions. Furthermore, while we did not find clear associations between FC and lesion location, it should be noted that 75% of patients who presented with pre-operative or post-operative FC impairment had lesions involving the frontal lobe, in particular in the right hemisphere. The association between the extent of the tumor-related lesion and the course of cognitive functioning is still a topic of ongoing debate (37–39), and the present findings on FC impairments add a piece of information to be clarified in future studies on larger samples.

With regard to awareness of one’s own financial difficulties as reported by patients and by their caregivers, we found that both of them tend to overestimate the financial skills, recognizing almost an intact FC both in terms of self-assessment carried out by the patient and assessment made by their caregivers, typically family members. Furthermore, while in the control group the self-assessment and caregiver assessment of FC are strongly correlated with the NADL-F scores, this association is absent in the patient cohort. These results suggest an inability to accurately assess financial functioning on the part of both the patient and their caregivers, even in the face of objective difficulties. The total NADL-F score is not even associated with caregiver-rated functional independence (IADL) at either baseline or follow-up. This suggests that FC may not directly reflect everyday functional abilities, at least as perceived by caregivers. This further underscore the need to assess FC by using tests, rather than simply relying on what emerges from interviews, even if these are semi-structured, such as the NADL-F questionnaire.

This lack of awareness has great clinical and legal relevance: a patient with impaired FC who maintains insight is more likely to accept protective oversight (e.g., power of attorney, shared financial management). Conversely, a patient with impaired FC and a lack of awareness is placed at substantial risk of poor decision-making, financial exploitation, and inappropriate independent financial actions, thereby exposing their personal security and autonomy (25).

In conclusion, this preliminary investigation into FC in patients with brain tumors highlights the potential decline in this functional ability both before and after surgical tumor removal, with a trend toward a recovery within 3 months of surgery in cases of preoperative impairment. This decline is associated with cognitive decline rather than specific characteristics of the tumor lesion. Patients tend to overestimate their FC, and the same is true for their caregivers. As a result, these deficits may remain unrecognized, particularly in the pre-operative phase, with potential significant consequences for the patient’s functional independence and legal protection.

### Limitations

This study presents limitations primarily related to the small and heterogenous clinical sample. This limits the statistical power of the analyses. This limitation is consistent with the inherently exploratory nature of the investigation, which aimed to examine, for the first time to our knowledge, how FC changes when brain tumors develop and are removed. The clinical heterogeneity concerns both the presence of neoplastic conditions of different etiologies, which may have influenced the results and hindered the identification of specific predictors of FC deficits depending on the type of brain tumor, considering their different clinical trajectories. Moreover, there was an imbalance with respect to the hemispheric side of the lesion, with a higher number of patients presenting right-sided damages, likely due to a selection bias. In fact, left brain-damaged patients with severe language impairments were excluded from recruitment because they were unable to undergo testing due to their language deficits. Future studies in large samples are needed not only to confirm the present results, but also to explore whether differences in FC are attributable to damage to specific mechanisms linked to the type, location, and stage of the tumor.

Another limitation lies in the structure of the NADL-F, which does not provide a cut-off value for the total score and, consequently, a normative threshold for overall FC. It is also worth noting that pathological (below cut-off values) scores were obtained in some NADL-F subtests not only by patients, but also by some healthy controls.

Finally, an interesting future direction for the study will be to incorporate second-level neuropsychological tests to identify specific cognitive functions whose impairment is predictive of a decline of FC.

## Data Availability

The datasets presented in this study can be found in online repositories. The repository and accession number can be found at: https://doi.org/10.5281/zenodo.17882355.
